# TAK1 regulates the tumor microenvironment through inflammatory, angiogenetic and apoptotic signaling cascades

**DOI:** 10.18632/oncotarget.27606

**Published:** 2020-05-26

**Authors:** Scott A. Scarneo, Kelly W. Yang, Jose R. Roques, Alanna Dai, Liesl S. Eibschutz, Philip Hughes, Timothy A.J. Haystead

**Affiliations:** ^1^ Department of Pharmacology and Cancer Biology, Duke University School of Medicine, Durham, NC 27710, USA; ^2^ Lineberger Comprehensive Cancer Center, University of North Carolina at Chapel Hill, Chapel Hill, NC 27599, USA

**Keywords:** TAK1, breast cancer, therapeutic, inflammation

## Abstract

Transforming growth factor beta-activated kinase 1 (TAK1) has been implicated for its role in inflammatory signaling and as an important mediator of cellular apoptosis and necroptosis in various cell types. Our recent discovery of a first-in-class, potent and selective TAK1 inhibitor, takinib, represents a novel pharmacological tool to evaluate TAK1’s role in cancer. In this study we evaluated the potential therapeutic capacity of TAK1 inhibition on tumor growth and on tumor microenvironment remodeling. In a screen of 16 cancer cell lines, takinib in combination with tumor necrosis factor (TNF) was found to induce cell death (>20%) in 6 out of 16 cell lines. Furthermore, knocking out of TAK1 in MDA-MB-231 cells dramatically increased their sensitization to TNF-mediated apoptosis. *In vivo* xenographs of MDA-MB-231 TAK1^KO^ tumors displayed delayed tumor growth and increased overall survival compared to TAK1^WT^ controls. Histological and proteomic analysis of TAK1^KO^ tumors showed altered angiogenic signaling and inflammatory signaling via immune cells. Overall, these findings suggest that the targeting of TAK1 in immune mediated cancers may be a novel therapeutic axis.

## INTRODUCTION

The role of immune cells and inflammation has been widely associated with aggressiveness and survival rates in many tumors, wherein pro-inflammatory signaling within the tumor microenvironment stimulates tumor cell growth and metastasis [1]. Both the innate and adaptive immune system have been implicated in tumorigenesis and maintenance of solid tumors [2]. Specifically, tumor-associated macrophages (TAMs) have been shown to create an immense tumor burden in breast cancers, with greater TAM burden leading to poor disease prognoses [3].

TAMs can influence the tumor microenvironment through secretion of biologically active molecules that promote tumor growth, angiogenesis and metastasis while limiting critical anti-tumor immune responses (immunosuppression) [4, 5]. TAMs arise from circulating monocytes, which are recruited to the tumor microenvironment largely due to tumor cell chemokine secretion. Specifically, TNF has been implicated in immune cell migration into the tumor [6]. TAMs enhance the tumor microenvironment in large part due to hyper-activating nuclear factor kappa-light-chain-enhancer of activated B cell (NF-κB) signaling, leading to downstream pro-inflammatory, pro-survival and metastatic phenotypes [7, 8]. Cytokines such as IL-6 and TNF aid in tumor development by activating pro-survival signaling and anti-apoptotic pathways in cancer cells [9, 10]. Furthermore, TAMs enhance the tumor microenvironment through neovascularization via vasculature signaling mechanisms, such as VEGF and angiogenin [11]. The constant exposure to inflammatory signals not only enhances the survival/growth mechanisms in tumor cells, but also educates T and dendritic cells to adopt an immunosuppressive phenotype and aid in disease progression [12]. Immunotherapies aimed at “re-educating” immune cells to adopt tumor surveillance phenotypes have shown great promise in immune responsive tumors [13, 14]. Remodeling of TAM phenotypes have shown potential in reducing tumor burden and in sensitizing tumors to an alternative therapeutic axis. Thus, targeted therapies aimed at modifying TAMs as well as targeting pro-survival mechanisms in tumor cells represent a novel, double edged, therapeutic intervention.

A key signaling element in pro-survival/inflammatory response pathways is the protein kinase TAK1 (transforming growth factor β-activated protein kinase 1) [15]. Upregulation of TAK1 can be seen in up to 30% of breast cancers, where they enhance tumor burden through increasing activity of NF-kβ and mitogen-activated protein kinases (MAPKs), which are important for tumorigenesis and inflammation [16]. Therefore, inhibitors of TAK1 constitute a means to block release of pro-inflammatory cytokines as well as cell migration. In addition to its anti-inflammatory properties, TAK1 has been shown to play an integral role in pro-survival/pro-apoptotic signaling [16–19]. Following TNF Receptor 1 activation by TNF, TAK1 stimulates downstream signaling cascades activating inflammatory and pro-survival proteins NF-kβ, cJun, JNK and p38 [16]. In contrast, it have been shown that inhibition of TAK1 in the presence of TNF induces caspases 3, 7 and 8 activation leading to apoptosis [16].

The duality of TNF stimulation makes TAK1 a key target in mediating between growth and apoptosis in cancer cells. With inhibition of TAK1 via small molecule inhibitors, we are able to bypass the inflammatory action of TNF while isolating and harnessing the apoptotic capabilities of TNF. Our recent discovery of the takinib scaffold has identified a potent, highly specific inhibitor of TAK1 (IC_50_ 9.5 nM) that we hypothesize can act as a novel small molecule therapy against immune responsive tumors. The present study investigates the therapeutic potential of takinib as an anti-cancer therapy as well as the role of TAK1 in inflammatory signaling within the tumor microenvironment.

## RESULTS

### Acute TNF exposure induces TAK1 mediated apoptosis in various cancer cell lines

**Figure 1:**
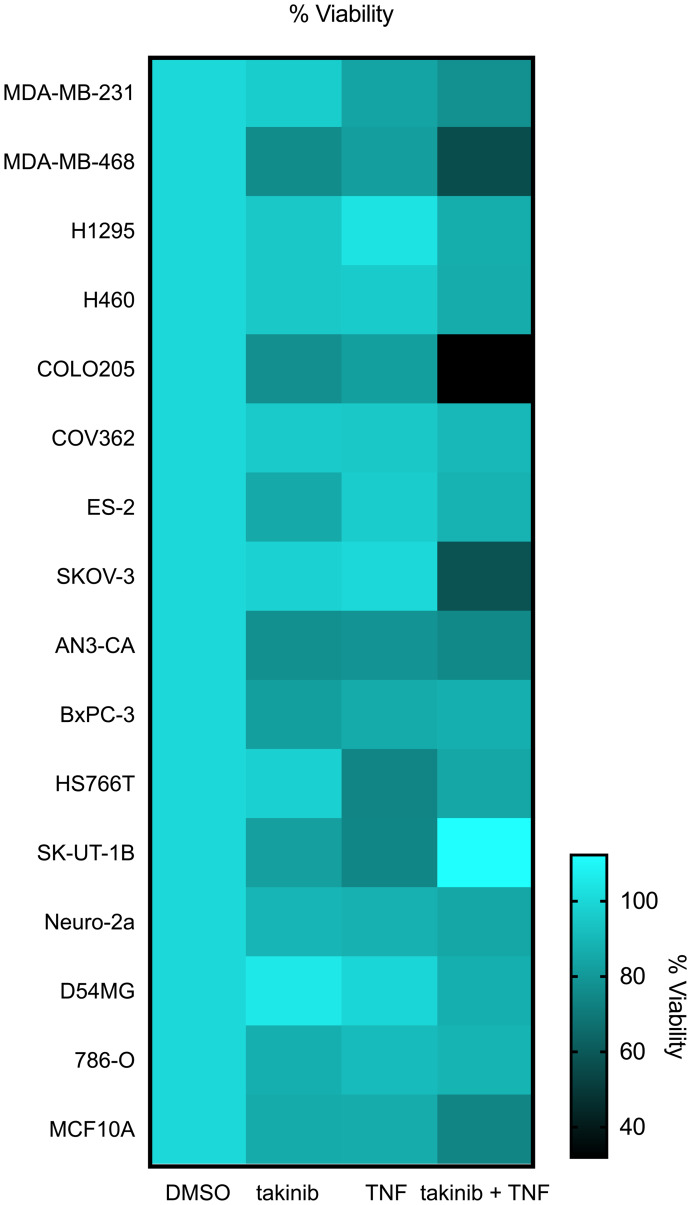
The anti-cancer effects of takinib *in vitro* is shown across various cell lines. Cells were plated at 80-90% confluency, serum starved for 24 hours, and evaluated 24 hours post treatment for percent cell death of takinib, takinib + TNF treatments; cell lines grouped by tissue.

We first investigated the effects of TAK1 pharmacological inhibition in 16 cell lines from various cancer tissues. Following 24 hours of TAK1 inhibition with takinib, minimal cell death was found in most cell lines (Figure 1). However, previous groups, including ours, have shown that TAK1-induced cell death is mediated by TNF signaling mechanisms [16, 20]. To further test this hypothesis, we treated cancer cells with takinib + TNF (30 ng/mL) combination therapy. We also examined the sensitivity of the cells to TNF alone and found that the combination therapy induced apoptosis (<80% cell survival) in 6 cell lines, compared to 3 by TNF alone. Furthermore, the data revealed cells that show initial sensitivity to TNF alone experience increased cell death after inhibition of TAK1 with takinib (Figure 1). We next tested the effects of takinib + TNF on MCF10A, a non-cancerous cell line. Cellular survival was not affected after the takinib + TNF combination therapy, with minimal cell toxicity observed at a 10 μM dosage of takinib (Figure 1).

**Figure 2:**
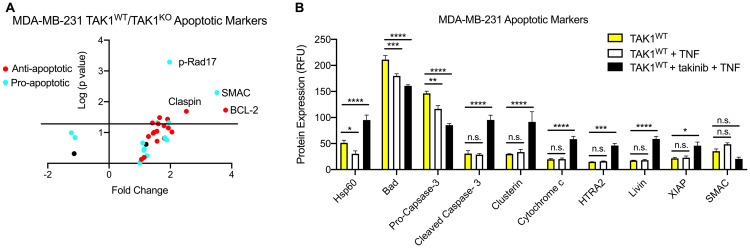
Both TAK1^KO^ and TAK1^WT^ cells treated with takinib + TNF are phenotypically distinguishable from TAK1^WT^. (**A**) Different levels of pro- and anti-apoptotic markers are found in MDA-MB-231 TAK1^WT^ vs TAK^WT^ treated with takinib + TNF. (**B**) TAK1^WT^ MDA-MB-231 cells were treated with either vehicle, TNF (30 ng/mL), or takinib (10 μM) + TNF for 12 hours. Compared to untreated TAK1^WT^ cells, takinib + TNF combination therapy saw an upregulation in apoptotic markers. Cleaved caspase-3, cytochrome c, HTRA2 and Livin levels were higher in takinib + TNF treated cells, compared to both vehicle and TNF only treatments. *n* = 3 ± SEM, Two-way ANOVA.

To further investigate the mechanisms behind takinib + TNF treatment on cancer cell death, we performed apoptosis marker screens on MDA-MB-231, a triple negative breast cancer line. Cells were treated with either vehicle, TNF (30 ng/mL), or takinib + TNF for 12 hours. In comparison to both vehicle and TNF only treatment, takinib + TNF treated cells saw an upregulation of apoptotic markers, including Hsp60 (*p <* 0.0001), cleaved caspase-3 (*p* < 0.0001), cytochrome c (*p* < 0.0001), HTRA2 (*p* < 0.0001), Livin (*p* < 0.0001), SMAC (P<0.006) and XIAP (*p* < 0.0001), two-way ANOVA (Figure 2A, 2B). Furthermore, takinib + TNF treatment of the colon cancer cell line, COLO205, showed robust induction of apoptotic proteins in response to TAK1 inhibition and TNF stimulation (Supplementary Figure 1). Upregulation of Cyto C, SMAC, and HTRA2 further support a TNF induced TAK1 mediated cell intrinsic apoptosis pathway [21].

### TAK1^KO^ MDA-MB-231 cells recapitulate effects of takinib

**Figure 3:**
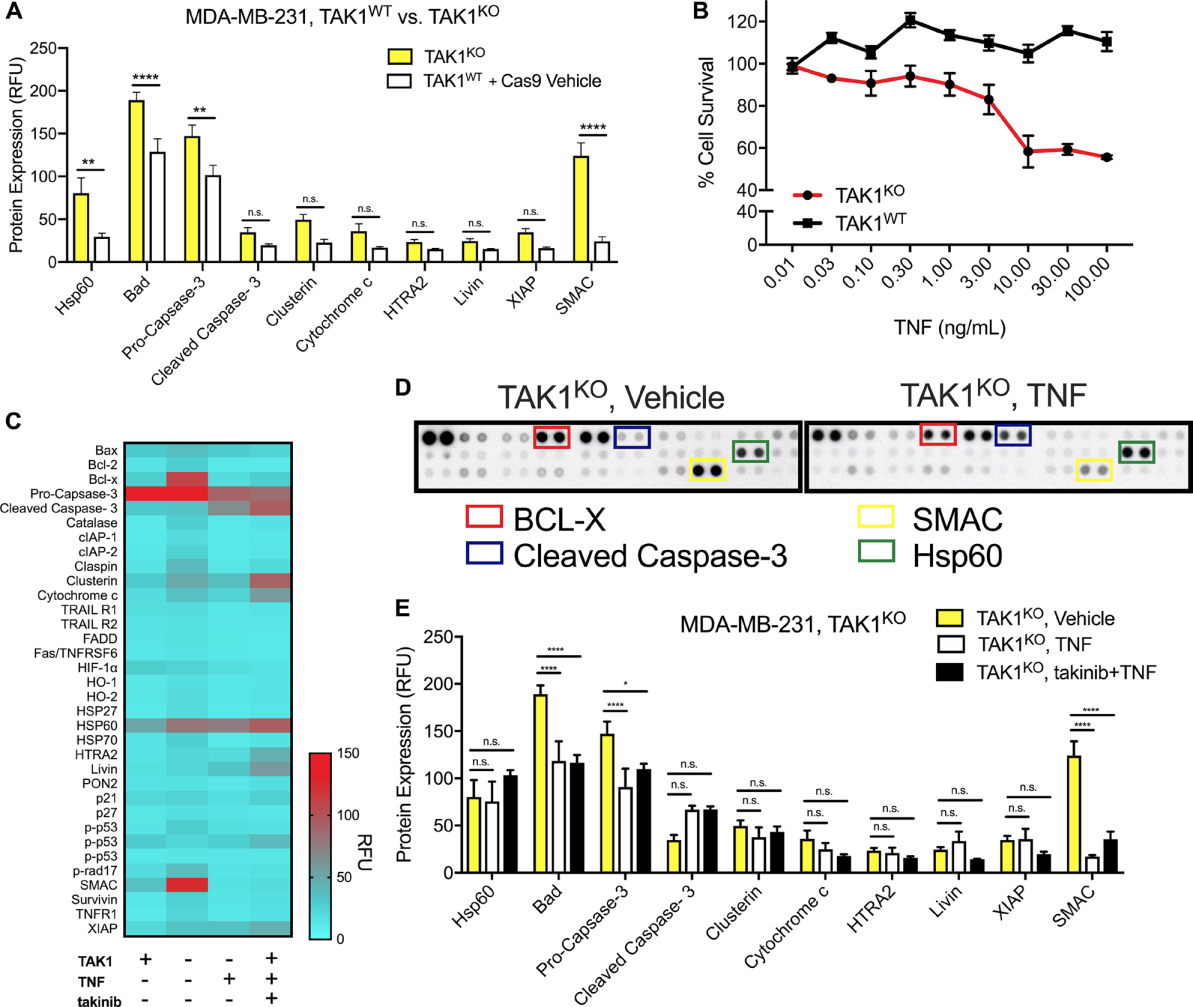
The efficacy of TAK1 inhibition on cell death is characterized by TAK1^KO^, as well as takinib + TNF treatment. (**A**) An apoptosis protein array revealed that in comparison to TAK1^WT^ (with Cas9 vehicle), TAK1^KO^ upregulated several apoptotic proteins. (**B**) Despite some similarities in apoptotic biomarkers, TAK1^KO^ were significantly more sensitive to apoptosis following TNF (30 ng/mL) treatments. (**C**) Apoptotic protein expression in TAK1mediated TNF apoptosis. (**D**) Differences in protein levels were evident in TAK1^KO^ vs. TAK1^KO^ with TNF proteome assays. (**E**) Compared to TNF and takinib + TNF treatments, TAK1^KO^ cells that were treated with vehicle, had significant upregulation of Hsp60, Cleaved Caspase-3 and SMAC. Two-way ANOVA.

Using the CRISPR Cas9 system, we created the TAK1^KO^ MDA-MB-231 cell line. Monoclonal TAK1^KO^ MDA-MB-231 clones were generated and TAK1^KO^ verified via western blot (Supplementary Figure 2). Even in their naïve states, TAK1^KO^ cells showed increased pro-apoptotic protein signatures, in comparison to TAK1^WT^, with upregulation of SMAC (*p* < 0.00001), Bad (*p* < 0.0001), Pro-Caspase-3 (*p* < 0.004) and Hsp60 (*p* < 0.01) (Figure 3A, Two-way ANOVA). We then asked if TAK1^KO^ would recapitulate the sensitivity to TNF (30 ng/mL) treatment. Here, we show that TAK1^KO^ cells show significantly higher sensitivity to TNF-induced apoptosis compared to TAK1^WT^ with an ED50 of ~0.5nM to TNF (Figure 3B).

Comparison of TAK1^WT^, TAK1^KO^, TAK1^KO^ + TNF, and TAK1^WT^ + takinib +TNF apoptotic profiles show both TAK1^KO^+TNF and TAK^WT^ + takinib + TNF show similar molecular apoptotic profiles resulting in cellular death (Figure 3C). We next compared the molecular signatures of TNF-induced cell death in TAK1^KO^ cells by treating cells with either vehicle, TNF only or takinib + TNF. Minimal differences were observed between TAK1^KO^ with TNF only and TAK1^KO^ with (takinib 10 μM + TNF) treatment, with predominately no changes seen in Hsp60 (*p* < 0.60), HTRA2 (*p* < 0.14), Livin (*p* < 0.14), SMAC (*p* < 0.99) and XIAP (*p* < 0.99), and a few significant changes in cytochrome c (*p* < 0.004) and cleaved caspase (*p* < 0.04), indicating that takinib selectively acts through TAK1, with no off target effects of TAK1^KO^+takinib observed (Figure 3D, 3E, Two-way ANOVA).

### TAK1^KO^ suppresses *in vivo* tumor growth

**Figure 4:**
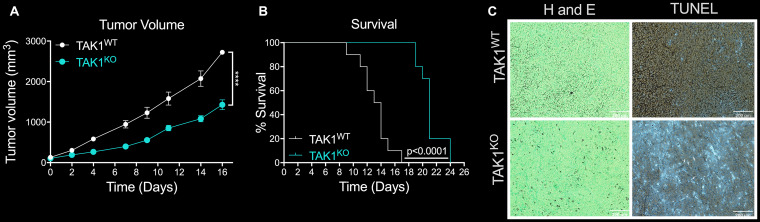
The efficacy of TAK^KO^ in inhibiting tumor growth and reducing survival rates *in vivo*. (**A**) Overall tumor growth was significantly reduced in mice injected with TAK1^KO^, in comparison to TAK1^WT^. Nude mice orthotopically injected with TAK1^WT^ cells with Cas9 control took on average 11 days to reach a tumor volume of 100 mm^3^, while it took TAK1^KO^ cells 14 days *n* = 10 ± SEM. (**B**) TAK1 ^KO^ injected mice outlived those injected with WT with Cas9 tumors *n* = 10 ± SEM. (**C**) Consistent with *in vitro* studies, TUNEL staining of tumors reveal increased cell death in TAK1^KO^ tumor slices in comparison to TAK1^WT^. Representative images *n* = 5, scale bar =200 μM.

To test the effects of TAK1 inhibition in tumor growth and overall survival, nude mice were orthotopically injected in the mammary fat pad with either MDA-MB-231 TAK1^WT^ with Cas9 control, or TAK1^KO^ cells. Days from injection to 100 mm^3^ tumor volume and survival time were evaluated. TAK1^KO^ tumors, on average, took 14 days to grow to 100 mm^3^, compared to 11 in TAK1^WT^ with Cas9 control (Supplementary Figure 3A). Furthermore, overall tumor growth was significantly reduced in TAK1^KO^ compared to TAK1^WT^ (*p* < 0.001) (Figure 4A). These data translated to an overall increase in survival of KO treated mice (*p* < 0.0001) (Figure 4B). Cell death was evaluated by TUNEL staining of the tumors, which showed increased TUNEL + cells in TAK1^KO^ tumors; results are consistent with our cellular studies (Figure 4C).

Following *in vitro* cell assays showing increased cell death of MDA-MB-231 TAK1 inhibited cells to TNF, we next sought to test this hypothesis *in vivo*. We first determined the *in vivo* MTD of TNF delivered via tail vein injection. Mice were dosed 3 times a week at 10, 30, 60 and 100 μg/kg showed no significant weight change (Supplementary Figure 3B). We next tested the ability of exogenous TNF to increase *in vivo* death of MDA-MB-231 TAK1^KO^ cells. At 100 mm^3^ tumor volume, TAK1^KO^ tumor bearing mice were treated with either TNF (30 μg/kg) 3 times a week or vehicle. Surprisingly, we saw no significant effect of TNF *in vivo* on tumor growth nor on survival (Supplementary Figure 3C, 3D).

### Takinib reduces TAM contribution of angiogenetic signaling

**Figure 5:**
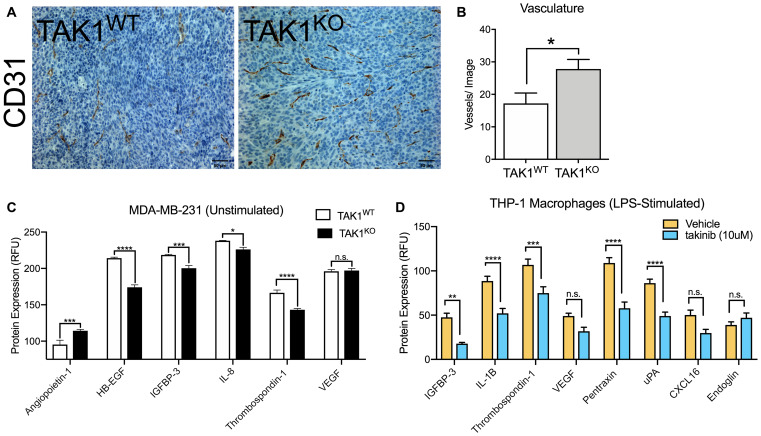
The role of TAK1 in cancer cell and macrophage vasculature. (**A**) Vasculature is increased in the MDA-MB-231 TAK1^KO^, in comparison to TAK1^WT^. (**B**) Vasculature was quantified by counting blood vessels; researcher was blinded. (**C**) Angiogenesis factors profile for MDA-MB-231 TAK1^WT^ vs TAK1^KO^ and for (**D**) LPS stimulated THP-1 vehicle vs takinib. RFU = Relative fluorescent unit. *n* = 5 ± SEM, ^*^ *p* = 0.05.

To further examine *in vivo* interactions between cancer and immune cells on tumor growth, we examined tumor slices from the previously mentioned mice injected with MDA-MB-231 TAK1^WT^ with Cas9 control or TAK1^KO^ cells. Previous studies, by our lab and others, have shown that TAK1 plays an important role in mediating inflammatory and potentially angiogenetic signaling cascades [22–24]. To test whether TAK1 mediated angiogenetic signaling was present in the tumor microenvironment, we stained tumor slices for CD31 (PECAM) expression. In comparison to TAK1^WT^, the TAK1^KO^ tumors showed increased vasculature (Figure 5A, 5B). We next profiled MDA-MB-231 TAK1^WT^, and TAK1^KO^ cells for angiogenesis factors. Minimal significant biological changes were seen between TAK1^WT^ and TAK1^KO^ cells with a 1.19 fold increase in angiopoietin-1 and decreases in expression of HB-EGF (1.23-fold), IGFBP-3 (1.09-fold), IL-8(1.05-fold) and thrombospondin (1.16-fold), suggesting that the TAK1 mediated angiogenetic signaling may occur outside of the cancer cells themselves (Figure 5C). Due to the large contribution of TAMs in the tumor immune microenvironment of many solid tumors, we also tested the ability of TAK1 inhibition to alter angiogenetic signaling in macrophages. THP-1, a human macrophage cell line, was stimulated with LPS, followed by treatment with either vehicle or takinib for 24 hours. Angiogenetic markers were profiled. Significant differences can be observed between the two treatments with a reduction of protein expression of 2.67-fold IGFBP-3 (*p* < 0.0012), 1.7-fold IL-1B (*p* < 0.0001), 1.42-fold thrombospondin (*p* < 0.0005), 1.54-fold VEGF (*p* < 0.156), 1.88-fold Pentraxin (*p* < 0.0001), 1.75-fold uPA (*p* < 0.0001), 1.68-fold CXCL16 (*p* < 0.056) seen (Figure 5D).

### Takinib reduces TAM contribution of inflammatory immune signaling

**Figure 6:**
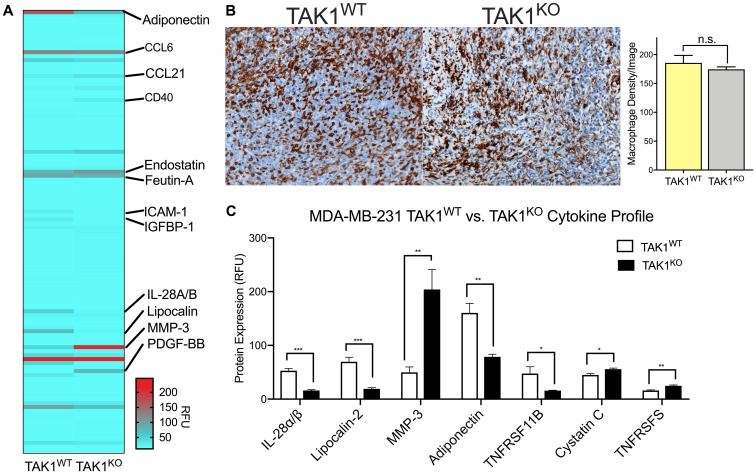
TAK1 changes cytokine expression of THP-1 macrophages without affecting their growth rates. (**A**) Tumors isolated from MDA-MB-231 TAK1^WT^ and TAK1^KO^ xenografts were profiled for cytokine and chemokine molecular expression. (**B**) TAK1 does not affect growth of THP-1 macrophages, suggesting macrophages are phenotypically changing their cytokine profiles. (**C**) TAK1 seems to play a role in cytokine expression, upregulating some and downregulating others. *n* = 4 ± SEM/WT, *n* = 4 ± SEM/TAK1^KO^ group. RFU = Relative fluorescent unit. ^*^ *p* < 0.05, ^**^ *p* < 0.01, ^***^ *p* < 0.0001, Student’s *T* test.

Aside from promoting angiogenesis, TAMs have also been known to increase inflammation in tumor microenvironments, activating pro-inflammation and pro-survival pathways. Our lab has previously shown takinib to successfully reduce cytokine and chemokine secretion in THP-1 human macrophages, following LPS inflammatory stimulation [15]. Here, we performed a cytokine screen of MDA-MB-231 TAK1^WT^ and TAK1^KO^ tumors. Similar to THP-1 macrophages, IL-28A/B (*p* < 0.0001) and lipocalin-2 (*p* < 0.0011) were significantly reduced in TAK1^KO^ cells compared to TAK1^WT^ controls (Figure 6A, 6C). However, Cystatin C (*p* < 0.015), CD40/TNFRSFS (*p* < 0.002) and MMP-3 (*p* < 0.007) were upregulated in TAK1^KO^ (Figure 6C).

To address whether changes in cytokine production are a consequence of altered TAM infiltration or changes in phenotype we next stained TAK1^KO^ and TAK1^WT^ tumors against F4/80 and quantified the number of macrophages present in the tumor stroma (Figure 6B). No significant changes were seen between the density of macrophages in TAK1^WT^ versus TAK1^KO^ tumors.

## DISCUSSION

Here we show the ability of takinib to not only induce TAK1 mediated apoptosis in TNF sensitive cancer cells, but to reduce pro-inflammatory and pro-angiogenic signaling in surrounding TAMs. TAK1 has been seen to play an integral role in mediating many signaling cascades including inflammatory, angiogenic and apoptotic; however, lack of selective and potent TAK1 pharmacological inhibitors have limited research around this target as a novel therapeutic axis. Here, we demonstrate the role of TAK1 in mediating TNF signal cascades in various cancer cell lines. Although TNF is widely considered a pro-inflammatory/survival signaling cytokine, we show that TAK1 inhibition blocks traditional TNF inflammatory and survival signaling and induces apoptosis in TAK1 dependent cancer cell lines.

In an effort to further validate TAK1 mediation of TNF survival-apoptosis signaling, we developed a TAK1^KO^ MDA-MB-231 cell line. TAK1^KO^ recapitulated the effects of TAK1 inhibition, further validating the role of this kinase in TNF signaling and apoptosis. In murine models, these knockout effects translated to tumor growth suppression. These results follow our knowledge of the molecular mechanisms of TAK1 inhibition. When TAK1 is not present, it cannot activate subsequent signaling pathways and is unable to promote cell proliferation. Interestingly we also noticed distinct cytokine profiles in the TAK1 KO tumors. Some of the differentials in cytokine expression between TAK1WT and TAK1KO cells can be used to explain the tumor suppression in the knockouts. For example, Endostatin which is present at higher concentrations in the TAK1KOs, suppresses both tumor growth and angiogenesis [25]. On the other hand, TAK1WTs express higher levels of Fetuin A, Lipocalin 2 and Reg3G, all of which promote tumorigenesis and angiogenesis [26–28].

At the time of writing, takinib demonstrated poor pharmacokinetics and bioavailability with rapid plasma clearance, limiting the ability to perform *in vivo* pharmacological studies with takinib. Further chemical modifications to the takinib scaffold such as the addition of solubilizing groups in the solvent accessible carbon positions of the aminobenzamide may improve bioavailability and provide a novel compound to evaluate TAK1’s role *in vivo*. However, despite lack of a pharmacological *in vivo* intervention, studies with TAK1^KO^ cell lines showed significantly reduced tumor burden and increased survival, with changes in inflammatory cytokines present in the tumor microenvironment.

Due to TAK1’s ubiquitous expression in most cells in the body, its pharmacological mechanism of action can be quite complex in certain disease contexts, such as cancer. It plays an integral role in inflammatory signaling of leukocyte populations, as well as angiogenic regulation associated with canonical inflammatory signaling [29]. However, previous studies as well as the one herein, have shown in certain cancer cell types TAK1 inhibition induces apoptosis, switching the cell fate from survival to apoptosis [30, 16]. There lies the potential that in conjunction with immunotherapy treatments, which stimulate dormant immune suppressive immune cell populations in the tumor immune microenvironment, one may further exacerbate immune cancer clearance by induction of endogenous apoptotic pathways from the TAK1-TNF signaling axis.

Additionally, often genetic instability in the cancer cells allow for mutations to occur, leaving the cells immune to the kinase inhibitor effects or induction of compensatory mechanisms. However, therapies targeting TAK1 appear to have effects not only at the cancer cell level but additionally at modulating the immune microenvironment. Non-cancerous immune cells may provide a more stable “druggable” cell population with TAK1 inhibitors targeting the chronic inflammatory processes that provide a positive feedback loop in tumors, promoting cell proliferation and metastasis. Thus, in the case of highly pro-inflammatory and aggressive cancers TAK1 inhibition may reduce the inflammatory signaling milieu which aids in the pro-survival/growth phenotype associated with highly aggressive cancers. Overall, here we have shown the role of TAK1 in both various cancer cells and TAM populations, showing a potential therapeutic axis in modulating the immune microenvironment of tumors.

## MATERIALS AND METHODS

### Animal care

Female nude mice were bred in-house or purchased from The Jackson Laboratory (Bar Harbor, ME, USA). All experiments were approved and carried out in accordance of the University of North Carolina-Chapel Hill, Institution Animal Care and Use Committee (IACUC) and conformed to the National Institutes of Health Guide for the Care and Use of Laboratory Animals. Mice were housed in a temperature and humidity-controlled facility under 12-hour light/dark cycle (lights on at 7 am) and access to food and water *ad libitum*.

### Tumor injections

Cells were mixed 1:1 with matrigel solution prior to injection into the mammary fat pad of female nude mice. Tumors were allowed to develop, and caliper measurements were obtained throughout the study period.

### Cell culture

THP-1, MDA-MB-231, COLO 205 and all other cells were obtained from Duke CCF. All cells are tested for mycoplasma and authenticated via Duke CCF prior to use. Cells were incubated at 37° C in 5% CO_2_. THP-1 was cultured in RPMI 1640×, 10% FBS, 1% Penicillin-Streptomycin (PS), HEPES, Pyruvate, Glucose and BME. All cell lines were cultured according to ATCC media guidelines.

### Macrophage differentiation

THP-1 cells were treated with 100 nM phorbol 12-myristate 13-acetate (PMA) for 72 hours in RPMI 1640× media. Cells were rested in PMA free media 48 hours prior to treatments. LPS (10 ng/mL) and IFNγ (50 ng/mL) were used for pro-inflammatory stimulation.

### Apoptotic, cytokine and chemokine arrays

THP-1 cells were differentiated as previously described in this manuscript. Following differentiation, cells were treated with 10 μM Takinib or DMSO vehicle control. 24 hours after treatment, supernatant was added to Human Cytokine XL proteome array (R&D Systems), or Angiogenesis proteome array. Apoptosis biomarkers were visualized with the Apoptosis Array kit (R&D Systems). All procedures were conducted in accordance with manufacturer protocol. Chemiluminescence was used to visualize protein quantities.

### Immunohistochemistry

Briefly, 10 μM cut parrafin embedded tumor slices from TAK1^KO^ or ^WT^ tumors underwent antigen retrieval prior to H&E and TUNEL staining as per manufacturer protocol.

### TAK1 gene editing

MDA-MB-231 cells were infected with CRISPR/CAS9 constructs with a blasticidin resistance gene. Cells were treated with blasticidin for 5 days following transfection to select transfected cells. Following stable cas-9 expression cells were transfected with lentiguide-puromycin sgRNA targeting TAK1 gene sequence. The following sgRNA sequences were used: sgRNA 1- 5′ CTCACCGGCCGAAGACGAGG 3′; sgRNA 2- 5′ CGACTACAAGGAGATCGAGG 3′; sgRNA 3- 5′ CATCTCACCGGCCGAAGACG 3′.

### Western blot analysis

Cells were lysed (50 mM Tris, 150 mM NaCl, 1 mM EDTA, 1% Triton-X100, 1 mM DTT, cOmplete protease (Roche) and PhosSTOP phosphatase inhibitor (Roche)) after indicated treatment and run on Criterion XT Tris-HCl gel 4%–15% gradient (Bio-Rad). Following transfer to PVDF membrane and blocking in 5% non-fat dry milk in TBST, membranes were incubated with antibody overnight. After incubation with secondary antibody, chemiluminescence was used to visualize bands.

### Drug treatment and cell viability assays

Cells were plated at 80% confluency on day one in media containing 10% FBS. After a 24 h incubation period, cells were pre-treated with either FBS-free media or varying doses of takinib in FBS-free media for 24 h prior to the addition of TNF (300 ng/mL). Cell viability assay was completed 24 h post-treatment and cell death quantified using Cell Titer Glo 2.0 (Promega) according to the manufacturer’s protocol.

### Quantification and statistical analysis

GraphPad Prism 8 was used for statistical analysis of viability, proteome assays, TAK1^KO^ analysis, survival analysis. For each analysis, total n and SEM are presented in the figure legend. Curves were plotted using variable slope (four parameters) non-linear fit. An alpha of 0.05 was used for all statistical analysis.

## SUPPLEMENTARY MATERIALS



## Abbreviations

TAM: tumor-associated macrophage
TAK1: Transforming growth factor β-activated kinase 1
NF-kβ: nuclear factor kappa-light-chain-enhancer of activated B cells
IRAK4: interleukin-1 receptor-associated kinase 4
RFU: relative fluorescent units
LPS: lipopolysaccharide
TNF: tumor necrosis factor

## References

[1] Elinav E, Nowarski R, Thaiss CA, Hu B, Jin C, Flavell RA Inflammation-induced cancer: crosstalk between tumours, immune cells and microorganisms. Nat Rev Cancer. 2013; 13:759–771. 10.1038/nrc3611. 24154716

[2] DeNardo DG, Barreto JB, Andreu P, Vasquez L, Tawfik D, Kolhatkar N, Coussens LM CD4(+) T cells regulate pulmonary metastasis of mammary carcinomas by enhancing protumor properties of macrophages. Cancer Cell. 2009; 16:91–102. 10.1016/j.ccr.2009.06.018. 19647220 PMC2778576

[3] Bingle L, Brown NJ, Lewis CE The role of tumour-associated macrophages in tumour progression: implications for new anticancer therapies. J Pathol. 2002; 196:254–265. 10.1002/path.1027. 11857487

[4] Binnewies M, Roberts EW, Kersten K, Chan V, Fearon DF, Merad M, Coussens LM, Gabrilovich DI, Ostrand-Rosenberg S, Hedrick CC, Vonderheide RH, Pittet MJ, Jain RK, et al Understanding the tumor immune microenvironment (TIME) for effective therapy. Nat Med. 2018; 24:541–550. 10.1038/s41591-018-0014-x. 29686425 PMC5998822

[5] Condeelis J, Pollard JW Macrophages: obligate partners for tumor cell migration, invasion, and metastasis. Cell. 2006; 124:263–266. 10.1016/j.cell.2006.01.007. 16439202

[6] Zijlmans HJ, Fleuren GJ, Baelde HJ, Eilers PH, Kenter GG, Gorter A Role of tumor-derived proinflammatory cytokines GM-CSF, TNF-alpha, and IL-12 in the migration and differentiation of antigen-presenting cells in cervical carcinoma. Cancer. 2007; 109:556–565. 10.1002/cncr.22428. 17177206

[7] Pikarsky E, Porat RM, Stein I, Abramovitch R, Amit S, Kasem S, Gutkovich-Pyest E, Urieli-Shoval S, Galun E, Ben-Neriah Y NF-kappaB functions as a tumour promoter in inflammation-associated cancer. Nature. 2004; 431:461–466. 10.1038/nature02924. 15329734

[8] Su S, Liu Q, Chen J, Chen J, Chen F, He C, Huang D, Wu W, Lin L, Huang W, Zhang J, Cui X, Zheng F, et al A positive feedback loop between mesenchymal-like cancer cells and macrophages is essential to breast cancer metastasis. Cancer Cell. 2014; 25:605–620. 10.1016/j.ccr.2014.03.021. 24823638

[9] Ferrajoli A, Keating MJ, Manshouri T, Giles FJ, Dey A, Estrov Z, Koller CA, Kurzrock R, Thomas DA, Faderl S, Lerner S, O’Brien S, Albitar M The clinical significance of tumor necrosis factor-alpha plasma level in patients having chronic lymphocytic leukemia. Blood. 2002; 100:1215–1219. 10.1182/blood.V100.4.1215.h81602001215_1215_1219. 12149200

[10] Knupfer H, Preiss R Significance of interleukin-6 (IL-6) in breast cancer. Breast Cancer Res Treat. 2007; 102:129–135. 10.1007/s10549-006-9328-3. 16927176

[11] Barbera-Guillem E, Nyhus JK, Wolford CC, Friece CR, Sampsel JW Vascular endothelial growth factor secretion by tumor-infiltrating macrophages essentially supports tumor angiogenesis, and IgG immune complexes potentiate the process. Cancer Res. 2002; 62:7042–7049. 12460925

[12] Jie HB, Gildener-Leapman N, Li J, Srivastava RM, Gibson SP, Whiteside TL, Ferris RL Intratumoral regulatory T cells upregulate immunosuppressive molecules in head and neck cancer patients. Br J Cancer. 2013; 109:2629–2635. 10.1038/bjc.2013.645. 24169351 PMC3833228

[13] Curran MA, Montalvo W, Yagita H, Allison JP PD-1 and CTLA-4 combination blockade expands infiltrating T cells and reduces regulatory T and myeloid cells within B16 melanoma tumors. Proc Natl Acad Sci USA. 2010; 107:4275–4280. 10.1073/pnas.0915174107. 20160101 PMC2840093

[14] Buchbinder EI, Desai A CTLA-4 and PD-1 Pathways: Similarities, Differences, and Implications of Their Inhibition. Am J Clin Oncol. 2016; 39:98–106. 10.1097/COC.0000000000000239. 26558876 PMC4892769

[15] Scarneo SA, Mansourati A, Eibschutz LS, Totzke J, Roques JR, Loiselle D, Carlson D, Hughes P, Haystead TAJ Genetic and pharmacological validation of TAK1 inhibition in macrophages as a therapeutic strategy to effectively inhibit TNF secretion. Sci Rep. 2018; 8:17058. 10.1038/s41598-018-35189-7. 30451876 PMC6242965

[16] Totzke J, Gurbani D, Raphemot R, Hughes PF, Bodoor K, Carlson DA, Loiselle DR, Bera AK, Eibschutz LS, Perkins MM, Eubanks AL, Campbell PL, Fox DA, et al Takinib, a Selective TAK1 Inhibitor, Broadens the Therapeutic Efficacy of TNF-alpha Inhibition for Cancer and Autoimmune Disease. Cell Chem Biol. 2017; 24:1029–39.e7. 10.1016/j.chembiol.2017.07.011. 28820959 PMC5576570

[17] Sanna MG, da Silva Correia J, Ducrey O, Lee J, Nomoto K, Schrantz N, Deveraux QL, Ulevitch RJ IAP suppression of apoptosis involves distinct mechanisms: the TAK1/JNK1 signaling cascade and caspase inhibition. Mol Cell Biol. 2002; 22:1754–1766. 10.1128/MCB.22.6.1754-1766.2002. 11865055 PMC135597

[18] Omori E, Matsumoto K, Sanjo H, Sato S, Akira S, Smart RC, Ninomiya-Tsuji J TAK1 is a master regulator of epidermal homeostasis involving skin inflammation and apoptosis. J Biol Chem. 2006; 281:19610–19617. 10.1074/jbc.M603384200. 16675448 PMC1797070

[19] Sethi G, Ahn KS, Pandey MK, Aggarwal BB Celastrol, a novel triterpene, potentiates TNF-induced apoptosis and suppresses invasion of tumor cells by inhibiting NF-kappaB-regulated gene products and TAK1-mediated NF-kappaB activation. Blood. 2007; 109:2727–2735. 10.1182/blood-2006-10-050807. 17110449

[20] Morioka S, Broglie P, Omori E, Ikeda Y, Takaesu G, Matsumoto K, Ninomiya-Tsuji J TAK1 kinase switches cell fate from apoptosis to necrosis following TNF stimulation. J Cell Biol. 2014; 204:607–623. 10.1083/jcb.201305070. 24535827 PMC3926964

[21] Fulda S, Debatin KM Extrinsic versus intrinsic apoptosis pathways in anticancer chemotherapy. Oncogene. 2006; 25:4798–4811. 10.1038/sj.onc.1209608. 16892092

[22] Morioka S, Inagaki M, Komatsu Y, Mishina Y, Matsumoto K, Ninomiya-Tsuji J TAK1 kinase signaling regulates embryonic angiogenesis by modulating endothelial cell survival and migration. Blood. 2012; 120:3846–3857. 10.1182/blood-2012-03-416198. 22972987 PMC3488895

[23] Sakurai H Targeting of TAK1 in inflammatory disorders and cancer. Trends Pharmacol Sci. 2012; 33:522–530. 10.1016/j.tips.2012.06.007. 22795313

[24] Ninomiya-Tsuji J, Kajino T, Ono K, Ohtomo T, Matsumoto M, Shiina M, Mihara M, Tsuchiya M, Matsumoto K A resorcylic acid lactone, 5Z-7-oxozeaenol, prevents inflammation by inhibiting the catalytic activity of TAK1 MAPK kinase kinase. J Biol Chem. 2003; 278:18485–18490. 10.1074/jbc.M207453200. 12624112

[25] Walia A, Yang JF, Huang YH, Rosenblatt MI, Chang JH, Azar DT Endostatin’s emerging roles in angiogenesis, lymphangiogenesis, disease, and clinical applications. Biochim Biophys Acta. 2015; 1850:2422–2438. 10.1016/j.bbagen.2015.09.007. 26367079 PMC4624607

[26] Ochieng J, Nangami G, Sakwe A, Moye C, Alvarez J, Whalen D, Thomas P, Lammers P Impact of Fetuin-A (AHSG) on Tumor Progression and Type 2 Diabetes. Int J Mol Sci. 2018; 19:2211. 10.3390/ijms19082211. 30060600 PMC6121429

[27] Yang J, Moses MA Lipocalin 2: a multifaceted modulator of human cancer. Cell Cycle. 2009; 8:2347–2352. 10.4161/cc.8.15.9224. 19571677 PMC3381736

[28] Zhang MY, Wang J, Guo J Role of Regenerating Islet-Derived Protein 3A in Gastrointestinal Cancer. Front Oncol. 2019; 9:1449. 10.3389/fonc.2019.01449. 31921694 PMC6928188

[29] Ajibade AA, Wang HY, Wang RF Cell type-specific function of TAK1 in innate immune signaling. Trends Immunol. 2013; 34:307–316. 10.1016/j.it.2013.03.007. 23664135

[30] Singh A, Sweeney MF, Yu M, Burger A, Greninger P, Benes C, Haber DA, Settleman J TAK1 inhibition promotes apoptosis in KRAS-dependent colon cancers. Cell. 2012; 148:639–650. 10.1016/j.cell.2011.12.033. 22341439 PMC3291475

