# Supplementary material for: TAK1 regulates the tumor microenvironment through inflammatory, angiogenetic and apoptotic signaling cascades
Source: Oncotarget. 2020 May 26;11(21):1961–70. doi: 10.18632/oncotarget.27606 (PMC7260121; doi:10.18632/oncotarget.27606)
Supplement: Supplementary file 1 [file oncotarget-11-1961-s001.pdf]

TAK1 regulates the tumor microenvironment through inflammatory, angiogenetic and apoptotic signaling cascades

SUPPLEMENTARY MATERIALS

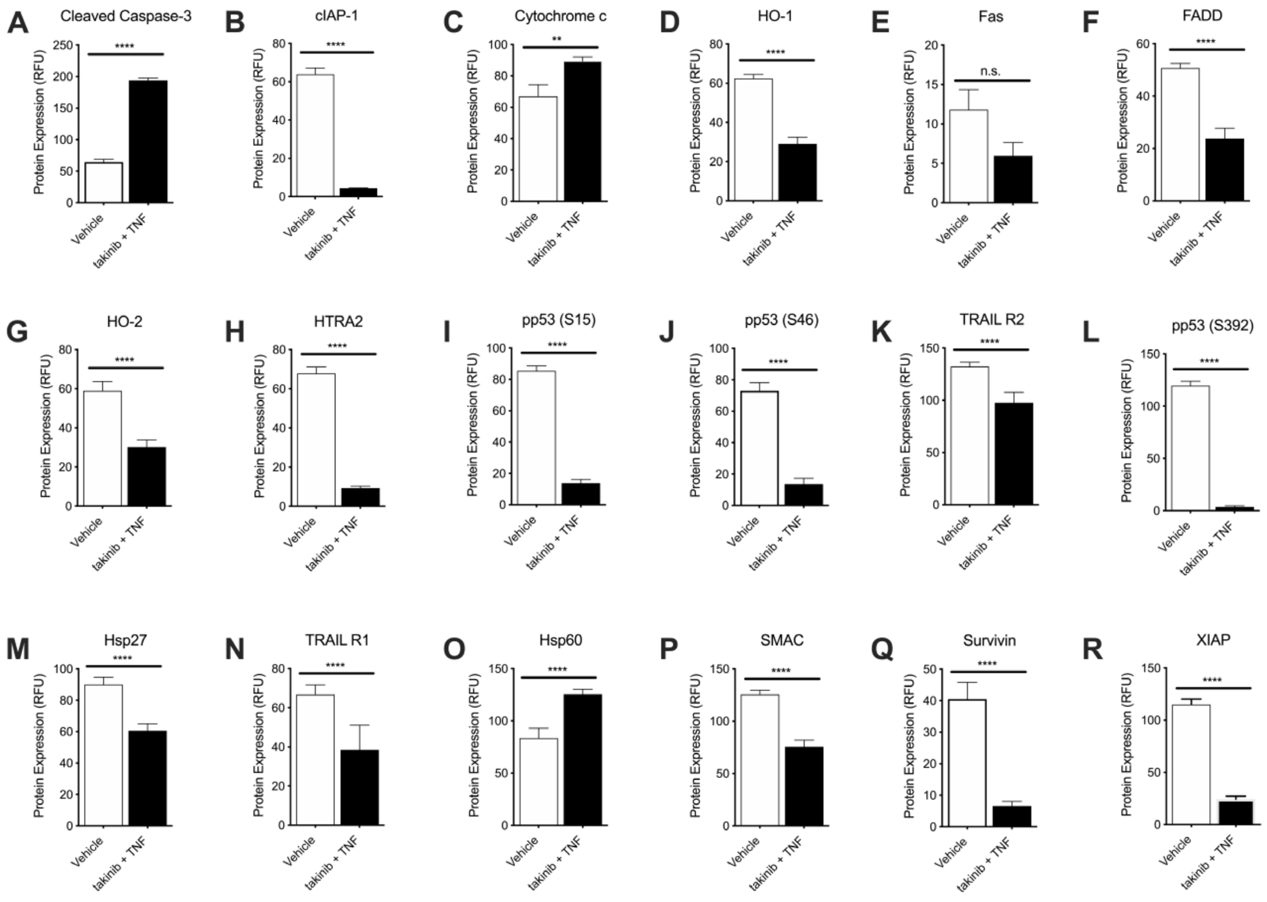

Supplementary Figure 1: Apoptotic biomarkers assay for COLO 205, a human colon cancer. (**A**–**R**) COLO205 was treated with either vehicle or takinib + TNF, then profiled for apoptotic biomarkers. $n = 4 \pm$ SEM for vehicle and takinib + TNF. RFU = Relative fluorescent unit. $^{*}p < 0.05$, $^{**}p < 0.01$, $^{***}p < 0.0001$, Two-way ANOVA.

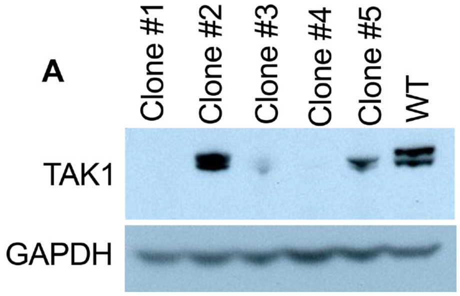

Supplementary Figure 2: Western blot of TAK1 expression in KO MDA-MB-231 clones.

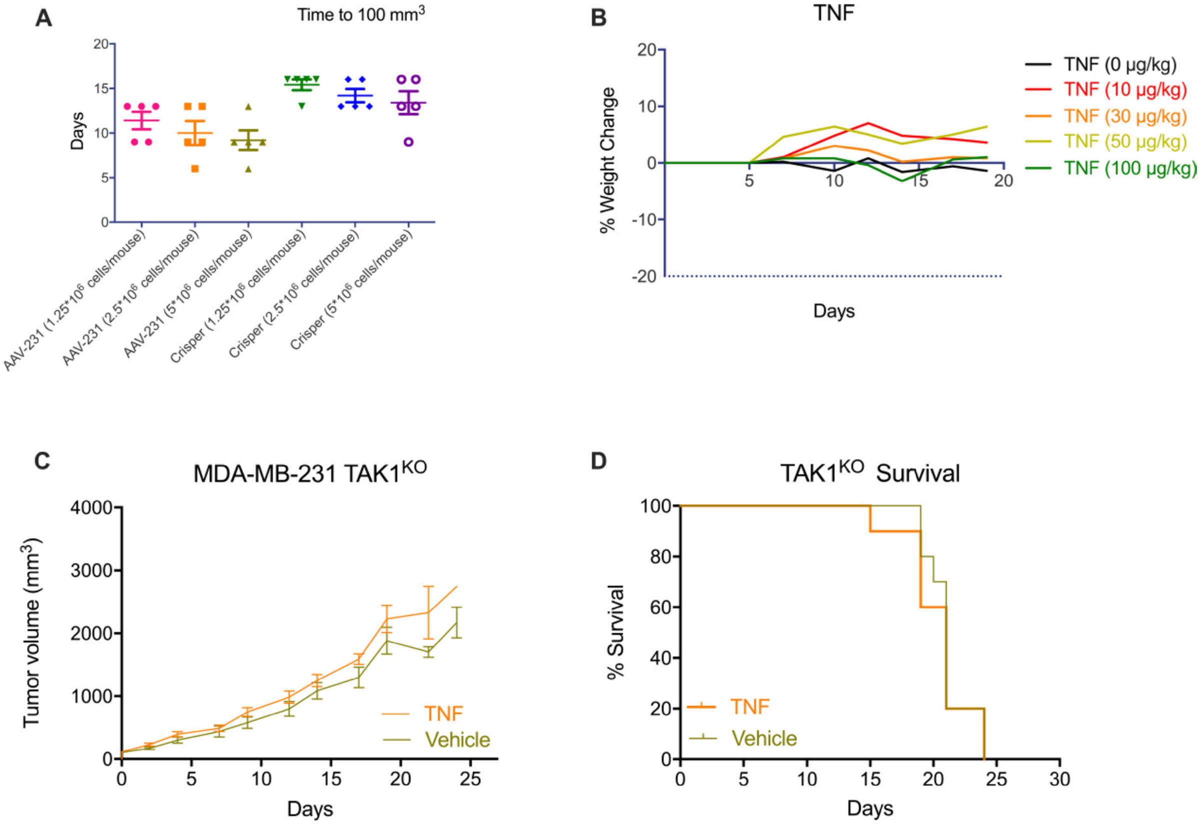

Supplementary Figure 3: Treatment with TNF was associated with faster tumor growth, weight loss and decreased survival in mice with TAK1^{KO} tumors. (**A**) Mice were injected with TAK1^{WT} or TAK1^{KO} cells, and the number of days it took *in vivo* tumors to reach a mass of 100 mm^3 was measured. (**B**) Varying concentrations of TNF treatment had different effects on weight change of injected mice. (**C**) Furthermore, amongst mice injected with TAK1^{KO} cells, TNF treatment can be seen to increase tumor volume, compared to vehicle treatment. (**D**) Mice injected with TAK1KO cells and treated with TNF had poorer survival rates than those treated with vehicle. $n = 12 \pm$ SEM.
